# Clinical utility of plasma Epstein-Barr virus DNA monitoring in pediatric Epstein‐Barr virus-associated hemophagocytic lymphohistiocytosis: a Chinese retrospective observational study

**DOI:** 10.1186/s13052-024-01689-8

**Published:** 2024-07-30

**Authors:** Wenzhi Zhang, Yuhong Yin, Ying Li, Li Cheng, Lizhen Zhao, Yun Peng, Xiaoyan Wu

**Affiliations:** Department of Pediatrics, Union Hospital, Tongji Medical College, Huazhong University of Science and Technology, Wuhan, 430022 China

**Keywords:** Epstein-Barr virus DNA, Hemophagocytic lymphohistiocytosis, Prognosis, Pediatric

## Abstract

**Background:**

Epstein-Barr virus DNA (EBV-DNA) is closely related to the pathogenesis and prognosis of EBV-associated hemophagocytic lymphohistiocytosis (EBV-HLH). The quantitative measurement of blood EBV-DNA is widely used in EBV-HLH, but there remains a lack of evidence to guide clinicians.

**Methods:**

A retrospective analysis was conducted on clinical manifestations, laboratory tests, 310 blood EBV-DNA loads, and prognosis of 51 pediatric patients diagnosed with EBV-HLH. Receiver operating characteristic (ROC) curves were utilized to determine the optimal cutoff values of EBV-DNA for predicting mortality and evaluating the active status of EBV-HLH.

**Results:**

EBV-positive- lymphoma-HLH had higher initial plasma EBV-DNA load(1.10 × 10^6^copies/ml) compared to the EBV-HLH group (1.98 × 10^4^ copies/ml) (*P =* 0.006), and experienced recurrently elevated plasma EBV-DNA levels during treatment. The optimal cut-off value of initial plasma EBV-DNA load in predicting mortality was 2.68 × 10^5^ copies/ml, with a sensitivity of 88.57% and a specificity of 56.25%. For determining the active status of HLH, the optimal cutoff value of PBMC EBV-DNA load during treatment was 2.95 × 10^5^ copies/ml, with a sensitivity of 69.14% and a specificity of 64.71%. The cut-off value of plasma EBV-DNA for determining active status was 1.32 × 10^3^ copies/ml, with a sensitivity of 84.34%, and a specificity of 87.67%. Patients with higher PBMC and plasma EBV-DNA at initial and those with repeated elevated plasma EBV-DNA during treatment had worse prognoses *(P* < 0.05).

**Conclusion:**

Dynamic monitoring of EBV-DNA is a valuable tool for assessing disease status and predicting the prognosis of EBV-HLH, with plasma EBV-DNA being more effective than PBMC EBV-DNA. Patients with high levels of PBMC and plasma EBV-DNA at initial and those with repeated elevated plasma EBV-DNA during treatment had worse prognoses.

## Introduction

Hemophagocytic lymphohistiocytosis (HLH) is a high-inflammatory syndrome caused by immunodeficiencies, characterized by fever, cytopenia, and markedly increased cytokines [1]. HLH can be classified as primary HLH and secondary HLH, with Epstein-Barr virus (EBV) associated-HLH being the most common type of secondary HLH [2]. The clinical presentation of EBV-HLH varies widely. Without timely and effective treatment, the 1-year overall survival rate of EBV-HLH is only 25% [3].

In addition to EBV-HLH, EBV infection can result in infectious mononucleosis, chronic active EBV infection (CAEBV), X-linked lymphoproliferative disease, nasopharyngeal carcinoma (NPC), and lymphoma. EBV-infected cells and/or cell-free EBV-DNA can be detected in the peripheral blood of patients with EBV-associated diseases. Consequently, the quantification of EBV-DNA load has been widely used as a noninvasive and convenient test for diagnosing and evaluating EBV-associated diseases [4].

Dynamic changes in EBV-DNA load have been shown to indicate the status of EBV-associated diseases, including post-transplant lymphoproliferative disorders (PTLD), NPC, and EBV-positive lymphoma. For instance, high EBV-DNA levels in peripheral blood mononuclear cells (PBMC) have been demonstrated to be associated with the development of PTLD [5]. In patients with NPC, plasma EBV-DNA has been identified as a sensitive and specific molecular marker, reflecting the disease stage, treatment response, and prognostic outcomes of NPC [6]. Moreover, patients with NK/T-cell lymphoma who test positive for plasma EBV-DNA before and after treatment have a higher likelihood of recurrence [7, 8].

A high initial EBV-DNA load in patients with EBV-HLH was related to poor treatment response and prognosis. However, the lack of a specific quantitative threshold for EBV-DNA load hampers clinical decision-making in the diagnosis and evaluation of HLH. Therefore, in this study, we retrospectively analyzed the clinical significance in prognosis and disease status determination of initial and dynamic changes of PBMC and plasma EBV-DNA in patients with EBV-HLH.

## Methods

### Patients

The study was carried out in accordance with the Declaration of Helsinki and approved by the ethics committee of the Union Hospital of Tongji Medical College, Huazhong University of Science and Technology (NO.2023 − 0253). Informed consent was waived due to the retrospective nature of the study.

This retrospective study enrolled 51 patients diagnosed with EBV-HLH admitted to Wuhan Union Hospital between January 2013 to December 2023, covering a period of 10 years. All patients met the HLH-2004 diagnostic criteria as well as the diagnostic criteria for EBV infection [9, 10]. The clinical characteristics, laboratory examinations, the results of 310 blood EBV-DNA tests (including 151 PBMC-EBV-DNA and 159 plasma EBV-DNA tests) were retrospectively analyzed. Additionally, results of 142 bone marrow (BM)- EBV-DNA tests were included in the analysis. Each patient had at least one PBMC-EBV-DNA or plasma EBV-DNA test result. Follow-up was conducted through outpatient clinic visits and telephone follow-up. The follow-up deadline was March 1, 2024 to assess patient outcomes and prognosis.

### Treatment and evaluation

According to therapy guidelines for HLH, the therapy strategy for patients with EBV-HLH included an etoposide-based protocol based on HLH-1994 and HLH-2004 protocols. Treatment response evaluations were performed at 2, 4, and 8 weeks of therapy.

The treatment response criteria are defined as follows: Complete response (CR) signifies the complete resolution of fever, serum ferritin levels, triglyceride, fibrinogen, soluble interleukin-2 receptor alpha chain(sCD25), and normalization of blood cell count. Partial response (PR) denotes a minimum 25% improvement in at least two symptoms/laboratory indicators. Relapse is characterized by the reappearance of fever accompanied by elevated serum ferritin levels and peripheral blood EBV-DNA copies. Progression was defined as the patient’s failure to achieve PR or worsening after achieving PR. Active status includes the following states: (1) Confirmed diagnosis status satisfying HLH diagnostic criteria; (2) Progression; (3) Relapse.

### EBV-DNA monitoring

EBV-DNA levels were monitored at the following time points: at the diagnosis and 2, 4, and 8 weeks after treatment initiation. Additional testing was carried out as deemed necessary by the clinician. The purpose of EBV-DNA testing extended beyond routine evaluations; it was employed to identify ambiguous disease conditions, such as a recurrence of fever, that couldn’t definitively be classified as either infections or relapses.

Commercial quantitative EBV PCR assay kit (Sansure Biotech Co. Ltd., Hunan, China) was used to quantify the viral load. The labeled lower detection limit for this assay was 400 copies/ml.

### Statistical analyses

Categorical variables were evaluated using a Chi-squared test or Fisher’s exact test. Quantitative variables were evaluated using Student’s t-test. Wilcoxon rank‐sum test was used for comparisons of continuous variables between two groups, and Mann–Whitney U or Kruskal‐Wallis tests were used for comparisons of continuous variables between multi‐groups.

To determine the diagnostic value of EBV-DNA load in plasma and PBMC, receiver operating characteristic (ROC) curves were constructed using GraphPad Prism version 8(GraphPad Software; LaJolla, CA). Statistics were performed using GraphPad Prism version. *P* values of < 0.05 were considered statistically significant.

## Results

### Baseline characteristics of pediatric EBV-HLH patients

Among the cohort of 51 pediatric patients diagnosed with EBV-HLH, 17.6% (9/51) patients were confirmed to have EBV-positive lymphoma following PET-CT imaging and subsequent pathology biopsy. The study group compromised 19 male patients (37.3%) and 32 female patients (62.7%), with a median age of 44 months (IQR: 25.5–85). The primary clinical manifestations observed in these patients with EBV-HLH included fever (100%), lymphadenopathy (51.0%), hepatosplenomegaly (90.2%), and multi-cavity effusion (45.1%)(Table 1).

**Table 1:** Descriptive baseline characteristics of patients (*n* = 51)

	*n* (%)
Age at diagnosis(months)	44 (25.5, 85)
gender	
Male	19(37.3%)
Female	32(62.7%)
EBV-positive-Lymphoma	9(17.6%)
Symptoms	
Fever	51(100%)
Lymphadenopathy	26(51.0%)
Hepatosplenomegaly	46(90.2%)
Multi-cavity effusion	23(45.1%)

The patients were divided into two groups based on their different backgrounds (EBV-HLH(*n* = 42), EBV-positive lymphoma-HLH(*n* = 9)). The PBMC and plasma EBV-DNA loads at the onset were compared among the two groups. The EBV-positive lymphoma-HLH group exhibited a significant higher plasma EBV-DNA load(1.10 × 10^6^ copies/ml) compared to the EBV-HLH group(1.98 × 10^4^ copies/ml) (*P =* 0.006), The difference in PBMC EBV-DNA load between the two groups was not statistically significant (*P* = 0.739)(Table 2).

**Table 2:** Initial EBV-DNA levels in patients with EBV-HLH with different backgrounds

	EBV-HLH(*n* = 42)	EBV positive lymphoma-HLH(*n* = 9)	*P*
PBMC EBV-DNA(copies/ml)	3.15 × 10^6^	1.08 × 10^7^	0.739
Plasma EBV-DNA(copies/ml)	1.98 × 10^4^	1.10 × 10^6^	0.006

### Prognostic value of initial EBV-DNA in EBV-HLH

**Fig. 1:**
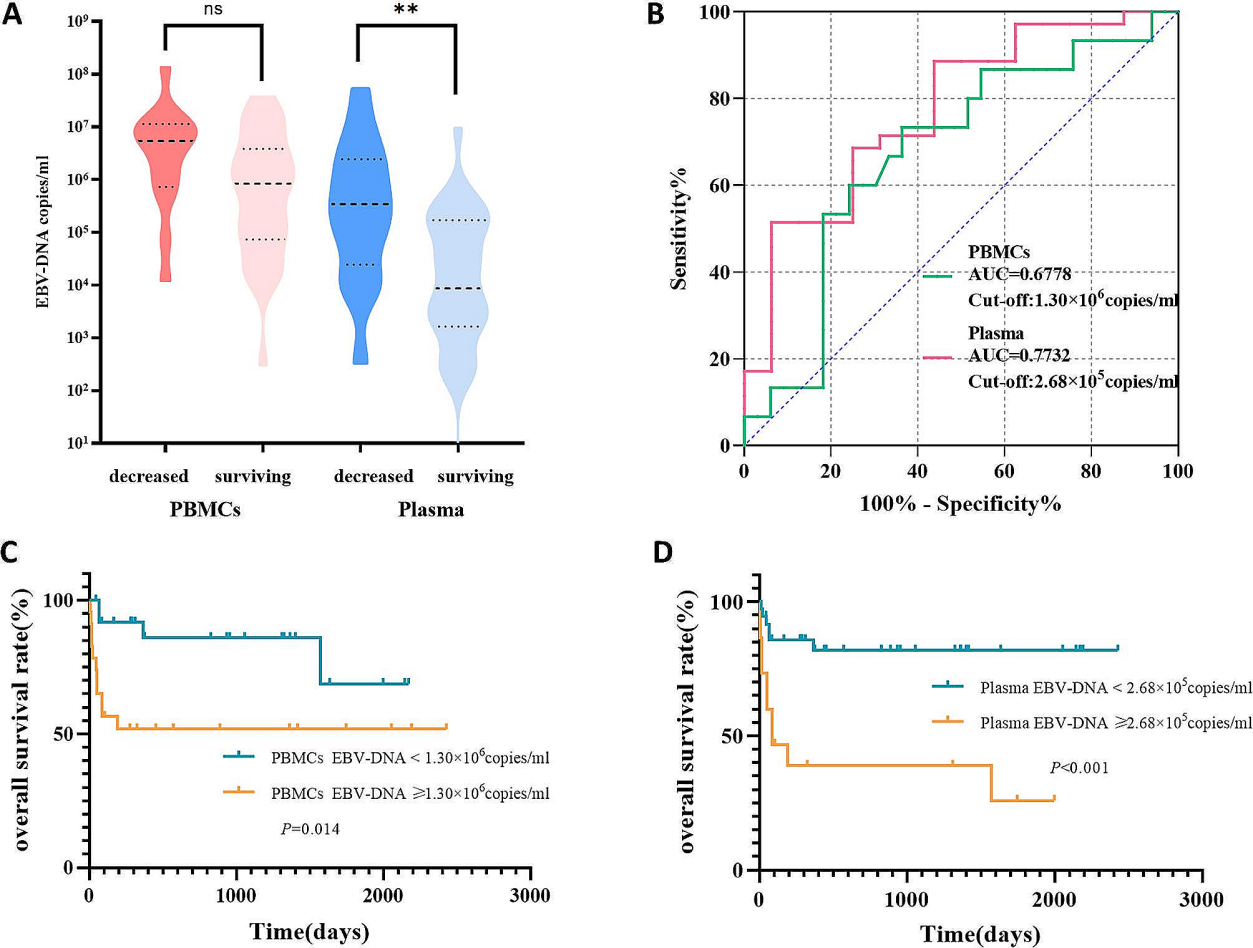
The significance of initial EBV-DNA in EBV-HLH. (**A**) EBV DNA loads in plasma and PBMCs in patients with EBV-HLH with different prognoses. (**B**) ROC curves for EBV DNA load in PBMCs and plasma of patients with EBV-HLH in the decreased group versus the surviving group. (**C**) Kaplan-Meier analysis of overall survival stratified by initial PBMCs EBV DNA load in patients with EBV-HLH. (**D**) Kaplan-Meier analysis of overall survival stratified by initial plasma EBV DNA load in patients with EBV-HLH

Based on the prognosis at follow-up, the patients were categorized into two groups: the deceased group, characterized by death resulting from disease progression, and the surviving group. It was observed that the levels of initial PBMC and plasma EBV-DNA were higher in the deceased group compared to the surviving group. Notably, a statistical difference was observed in the plasma EBV-DNA between the two groups (*P* = 0.002) (Fig. 1A).

ROC analysis was used to determine the optimal cutoff value for predicting the prognosis of EBV-HLH. The area under the curve (AUC) for predicting death based on EBV-DNA loads in PBMC was 0.6778 (0.5136 to 0.8420), with a cut-off value of 1.30 × 10^6^copies/ml, a sensitivity of 73.33%, and a specificity of 63.64% (Fig. 1B). Based on the cutoff value, the patients were divided into two groups: those with an initial PBMC-EBV-DNA ≥ 1.30 × 10^6^ copies/ml and those with an initial < 1.30 × 10^6^ copies/ml. The survival rates of the two groups were compared using the Log-rank test (Fig. 1C). Results revealed that patients with initial PBMC-EBV-DNA ≥ 1.30 × 10^6^copies/ml had a significantly lower overall survival (*P* = 0.014).

The AUC for predicting death based on EBV-DNA loads in plasma was determined to be 0.7732 (0.6344 to 0.9121), with an optimal cutoff value of 2.68 × 10^5^copies/ml, a sensitivity of 88.57%, and a specificity of 56.25%. Patients were divided into two groups based on initial plasma EBV-DNA levels: those with initial plasma EBV-DNA loads ≥ 2.68 × 10^5^copies/ml and those with loads < 2.68 × 10^5^copies/ml. The survival rates of these groups were compared using the Log-rank test, revealing that patients with initial plasma EBV-DNA levels ≥ 2.68 × 10^5^ copies/ml exhibited a significantly lower overall survival rate. (*P* < 0.001) (Fig. 1D).

### The significance of dynamic monitoring EBV-DNA for determining the disease status of EBV-HLH

**Fig. 2:**
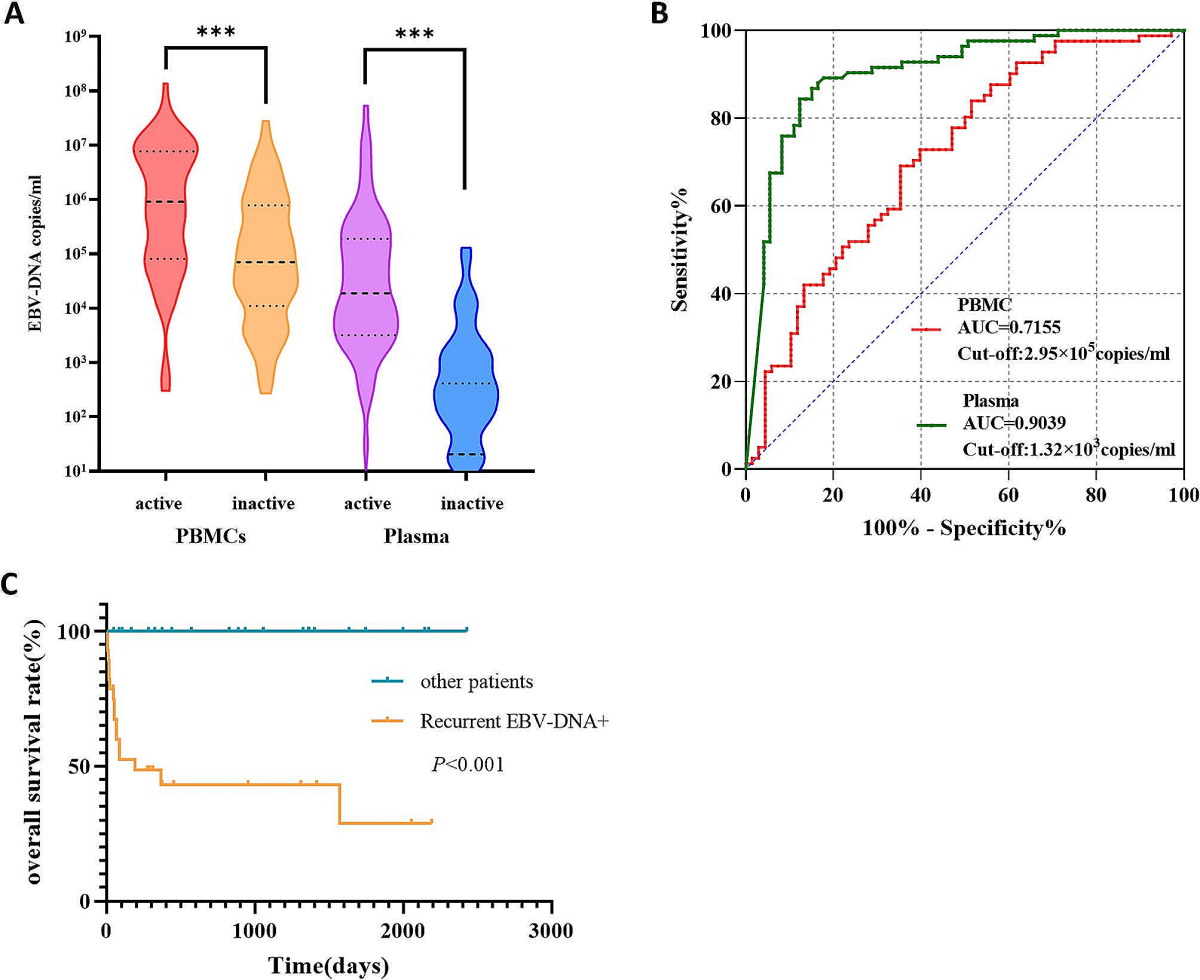
The significance of dynamic monitoring EBV-DNA in EBV-HLH. (**A**) EBV DNA loads in plasma and PBMCs in different activation states of patients with EBV-HLH. (**B**) ROC curves for EBV DNA loads in PBMCs and plasma of patients with EBV-HLH in active group versus inactive group. (**C**) Kaplan-Meier analysis of overall survival stratified by dynamic plasma EBV DNA trajectories in patients with EBV-HLH

In our study, a total of 51 patients underwent 151 PBMC-EBV-DNA tests and 159 plasma EBV-DNA tests. The 310 EBV-DNA test results were categorized into two groups: active and inactive, based on the patient’s disease status at the time of each test. The median PBMC-EBV-DNA load in the active group was 9.15 × 10^5^ copies/ml, which was significantly higher than that in the inactive group(7.04 × 10^4^ copies/ml) (*P* < 0.001). Similarly, the median plasma EBV-DNA load in the active group(1.87 × 10^4^copies/ml) was significantly higher compared with the inactive group(2.04 × 10^1^copies/ml) (*P* < 0.001) (Fig. 2A).

The levels of bone marrow EBV-DNA, white blood count(WBC), hemoglobin(HB), platelet count(PLT), alanine aminotransferase (ALT), aspartate aminotransferase (AST), and cytokines were compared in different disease states. It was observed that the blood cell counts(WBC, HB, PLT) in the active group were significantly lower compared to the inactive group, while the levels of bone marrow(BM) EBV-DNA, ALT, AST, and cytokines were significantly higher (*P* < 0.05) (Table 3).

To further assess the efficacy of PBMC and plasma EBV-DNA in distinguishing between active and inactive statues in EBV-HLH, we constructed an ROC curve (Fig. 2B). The results revealed an AUC of 0.7155 (95% CI: 0.6326 to 0.7984) for PBMC-EBV-DNA diagnosis of the active status. The cut-off value was determined to be 2.95 × 10^5^ copies/ml, with a sensitivity of 69.14%, and a specificity of 64.71%. Similarly, the AUC for plasma EBV-DNA diagnosis of active status was found to be 0.9039 (0.8541 to 0.9536), with a cut-off value of 1.32 × 10^3^ copies/ml, a sensitivity of 84.34%, and a specificity of 87.67%. Hence, both PBMC and plasma EBV-DNA demonstrate significant diagnostic value in distinguishing between active and inactive status in patients with EBV-HLH.

**Table 3:** laboratory results in the different activation status of patients with EBV-HLH

	Active	inactive	*P*
PBMC EBV-DNA(copies/ml)	9.15 × 10^5^	7.04 × 10^4^	< 0.001
Plasma EBV-DNA(copies/ml)	1.87 × 10^4^	2.04 × 10^1^	< 0.001
PBMC EBV-DNA in BM(copies/ml) BM(copies/ml)	5.69 × 10^6^	3.07 × 10^5^	0.005
Plasma EBV-DNA in BM(copies/ml) BM(copies/ml)	2.96 × 10^4^	1.50 × 10^3^	0.001
WBC(G/L)	3.0	4.5	0.005
HB(g/L)	90.2	100.9	< 0.001
PLT(G/L)	102.1	282.3	< 0.001
ALT(U/L)	172.3	44.0	< 0.001
AST(U/L)	292.1	40.8	< 0.001
LDH(U/L)	1598.4	330.8	< 0.001
IL-6(pg/ml)	92.1	10.7	0.001
IL-10(pg/ml)	439.0	30.3	0.001
IFN-γ(pg/ml)	319.0	30.3	0.032

### Prognostic value of dynamic monitoring EBV-DNA in EBV-HLH

During the treatment, we monitored blood EBV-DNA in EBV-HLH patients (including 151 of PBMC-EBV-DNA and 159 plasma EBV-DNA tests). Based on the results from dynamic monitoring of plasma EBV-DNA, the patients were divided into recurrent positive patients and other patients (those who tested negative after treatment and remained negative). One patient underwent only one EBV-DNA test before treatment and was therefore excluded from the analysis of this part. The rate of disease relapse in plasma EBV-DNA recurrent positive patients (85.2%) was significantly higher than that in other patients (4.3%) (*P* < 0.001). Furthermore, recurrent positive plasma EBV-DNA was observed in all nine EBV-positive-lymphoma-HLH patients. (Table 4).

**Table 4:** Comparison of relapse rates and disease backgrounds between patients with recurrent positive plasma EBV-DNA and other patients ^a^

	Plasma EBV-DNA recurrently/persisitantly positive(*n* = 27)	Plasma EBV-DNA persistently negative(*n* = 23)	*P*
Relapse/refractory(*n* = 24)	23(85.2%)	1(4.3%)	<0.001
Sustained remission (*n* = 26)	4(14.8%)	22(95.7%)	
Disease background			
EBV-HLH(*n* = 41)	18(43.9%)	23(56.1%)	0.002
EBV-positive-lymphoma-HLH(*n* = 9)	9(100%)	0(0%)	

a: One patient underwent only one EBV-DNA test before treatment and therefore was excluded from inclusion in this table.

To determine the correlation between dynamic changes of EBV-DNA and the prognosis of EBV-HLH patients, survival curves were plotted for both groups. It was observed that recurrent positive patients had a significantly lower overall survival rate than other patients (*P* < 0.001) (Fig. 2C).

## Discussion

EBV was the first human cancer virus, which was discovered in Burkitt lymphoma in the 1960s. It infects about 90% of adults worldwide [11, 12]. Quantitative detection of EBV-DNA by real-time PCR has been used for over 20 years [13]. The significance of quantifying EBV-DNA in EBV-related diseases has been extensively researched and studied.

The diagnostic value of EBV-DNA in PBMC and plasma varies among different EBV-related diseases. Different forms of EBV-DNA in peripheral blood are associated with diverse states in EBV-associated diseases. In PTLD, cells infected by EBV proliferate in lymphoid tissues and transit into the peripheral blood. Therefore, most of the EBV-DNA in the peripheral blood is cell-associated. Zhou et al. found that PBMC EBV-DNA was more effective than plasma EBV-DNA in predicting EBV-related PTLD and Graft-versus-host disease(GVHD) [5]. However, in Hodgkin’s lymphoma (HL) and NPC, most of the EBV-infected tumor cells remain within the tissue, and the EBV-DNA in peripheral blood is mainly found cell-free from apoptotic or necrotic cells [6, 14, 15]. Plasma EBV-DNA levels can reflect the tumor burden and cell damage caused by inflammation or immune injury in patients. Therefore, it can be used as a biomarker to evaluate disease severity or patient prognosis [16].

According to the results of the ROC curve analyses, we found that the AUC of plasma EBV-DNA was higher than that of PBMC EBV-DNA in predicting the prognosis and active status of EBV-HLH patients. This finding suggests that monitoring the condition of EBV-HLH patients through plasma EBV-DNA is more effective than using PBMC EBV-DNA, and it is consistent with previous studies [17, 18]. Nonetheless, the detection of PBMC-EBV-DNA is still meaningful. In EBV-HLH, EBV can infect B cells, T cells, and NK cells. Detecting PBMC EBV-DNA in different lymphocytes may help distinguish potential p-HLH and lymphoma [19]. Moreover, in EBV-HLH cases primarily involving B lymphocytes, rituximab has demonstrated efficacy in clearing EBV and alleviating symptoms. However, in EBV-HLH infecting T and NK lymphocytes, rituximab was unable to clear the virus effectively and could potentially lead to immune deficiency in patients [3, 20].

EBV-HLH patients progress rapidly and require a prompt determination of active status and timely treatment. We compared the levels of EBV-DNA detected in EBV-HLH patients in active state and inactive state. It was found that the EBV-DNA load in the active state was higher than that in the inactive state (Fig. 2A). The application of detectable EBV-DNA in plasma for diagnosing the active status in EBV-HLH resulted in a sensitivity of 84.34%, and a specificity of 87.67%. Similar findings had been reported in previous studies. MiaoZhen found that patients with EBV-NK-LPD who had plasma EBV-DNA levels exceeding 4.16 × 10^3^copies/ml were more likely to develop HLH [21]. Our results suggest that elevated plasma EBV-DNA levels in EBV-HLH patients should serve as an alert for active status and require timely treatment.

Patients with EBV-HLH often miss the optimal time for etiological diagnosis due to their critical condition at onset. In our study, a group of nine patients diagnosed with EBV-positive lymphoma exhibited significantly higher levels of plasma EBV-DNA at the initial stage compared to other patients. This observation may be attributed to the suppressed immune function commonly observed in tumor patients. Furthermore, all nine patients in our study experienced repeated positive plasma EBV-DNA during treatment and a significantly higher frequency of disease recurrence than other EBV-HLH patients. Therefore, patients with high initial plasma EBV-DNA load and repeated positive plasma EBV-DNA during treatment should consider the potential diagnosis of lymphoma. Such patients often require specialized therapy targeting lymphoma, and may eventually require hematopoietic stem cell transplantation [17, 22].

In our study, patients with PBMC EBV-DNA level ≥ 1.30 × 10^6^copies/ml and plasma EBV-DNA level ≥ 2.68 × 10^5^copies/ml at initial diagnosis exhibited poorer prognoses (Fig. 1CD). Some patients had a significant decrease or even clearance of plasma EBV-DNA after treatment, they subsequently exhibited repeat positivity for plasma EBV-DNA during further treatment. The survival rate of these patients was noticeably lower. Conversely, patients who maintained persistent decrease or clearance of plasma EBV-DNA during treatment demonstrated more favorable long-term prognoses (Fig. 2C). A study focusing on the L-DEP regimen of EBV-HLH patients revealed that the reduction in EBV-DNA loads at an early time point following therapy could not predict improved long-term outcomes [23]. EBV reactivation and high EBV-DNA load have been confirmed as risk factors for the occurrence of EBV-HLH [24]. In our study, patients with repeated positive results for plasma EBV-DNA exhibited a notably higher recurrence rate compared to those with consistently negative results. This increased recurrence rate may contribute to their lower survival rates. Similar observations have also been reported in NPC patients, where an increase in plasma EBV-DNA often indicates disease progression or recurrence [6]. In addition, the survival rate of NPC patients with persistent clearance of EBV-DNA is also significantly higher than that of patients without persistent clearance [25].

In conclusion, EBV-HLH remains a critical disease with a high mortality rate. Therefore, developing a new rapid indicator to monitor patients’ conditions is crucial for EBV-HLH. Based on our findings, we proposed a diagnostic strategy to differentiate EBV-HLH’s active state, which can aid in the evaluation of patients’ conditions. This strategy combines a cutoff EBV DNA load of 1.32 × 10^3^ copies/ml in plasma. Moreover, we propose monitoring of EBV-DNA levels, particularly in plasma, during treatment of EBV-HLH. Patients with high initial EBV-DNA load and repeat positive plasma EBV-DNA levels are likely to have a poor prognosis.

This research has several limitations. This study was conducted at a single center and had a small sample size, thus further verification is needed in larger sample sizes and multiple centers.

## Conclusions

Children with EBV-HLH present with rapid progression and significant prognostic variability. This study identified the significant value of EBV-DNA detection in both prognostic assessment and disease status determination among EBV-HLH patients. Furthermore, our research has established cutoff values for EBV-DNA to predict prognosis and disease status, with plasma EBV-DNA demonstrating superior efficacy in evaluating the disease status of EBV-HLH patients. This study provides insights for clinicians in their clinical decision-making processes concerning pediatric EBV-HLH patients.

## Data Availability

The datasets used and/or analyzed during the current study are available from the corresponding author on reasonable request.

## Abbreviations

AUC: Area under the curve
BM: Bone marrow
CAEBV: Chronic active EBV infection
CR: Complete response
EBV: Epstein-Barr virus
GVHD: Graft-versus-host disease
HLH: Hemophagocytic lymphohistiocytosis
NPC: Nasopharyngeal carcinoma
PBMC: Peripheral blood mononuclear cells
PR: Partial response
PTLD: Post-transplant lymphoproliferative disorders
ROC: Receiver operating characteristic
sCD25: Soluble interleukin-2 receptor alpha chain
